# Antimalarial Effect of the Root of *Silene macrosolen* A. Rich (Caryophyllaceae) on *Plasmodium*-*berghei*-Infected Mice

**DOI:** 10.1155/2021/8833865

**Published:** 2021-03-16

**Authors:** Gebru Hagos Atsbha, Rajkapoor Balasubramanian, Abadi Kahsu Gebre

**Affiliations:** ^1^Department of Pharmacy, College of Health Sciences, Adigrat University, Adigrat, Ethiopia; ^2^School of Pharmacy, College of Health Sciences, Mekelle University, Mekelle, Ethiopia; ^3^Department of Pharmacology, J.K.K. Nattraja College of Pharmacy, Komarapalayam-638 183, Tamilnadu, India

## Abstract

**Background:**

Malaria remains a major public health problem globally. Poor access to antimalarial drugs compounded with rapidly evolving drug resistance encourages researchers to continuously look for new drugs. Of importance, traditionally used medicines of plant origin are the highest priority as the ethnobotanical claim can be used as an important clue for its safety and efficacy profiles. *Silene macrosolen* A. Rich (Caryophyllaceae) has been traditionally used for malaria treatment in Ethiopia. Therefore, this study was aimed to evaluate the *in vivo* antimalarial activity of the plant against *Plasmodium*-*berghei*-infected (ANKA strain) Swiss albino mice.

**Methods:**

The dried powdered root of *Silene macrosolen* was extracted using 80% methanol. The crude extract was fractionated using chloroform, ethyl acetate, and distilled water that have different affinities to plant phytoconstituents. The *in vivo* antimalarial activities of the crude extract were evaluated using 4-day suppressive, prophylactic, and curative tests. The antimalarial activity of the solvent fractions was evaluated in a 4-day suppressive test. The oral acute toxicity of the crude extract was also determined according to the OECD guidelines.

**Results:**

The percentage of parasite suppression on the crude extract was 31.02%, 35.82%, and 39.23% in prophylactic, curative, and 4-day suppressive tests, respectively, at the tested dose level of 400 mg/kg. The percentages of chemosuppression of the solvent fractions (400 mg/kg) were 43.07%, 42.61%, and 38.38% in aqueous, ethyl acetate, and chloroform fractions, respectively. Both the crude extract and solvent fractions also significantly prolonged survival time except in the prophylactic test. In addition, prevention of weight loss and reduction in temperature and packed cell volume (PCV) were observed in crude extract as well as solvent fractions. The acute toxicity test of the plant extract also exhibited no sign of toxicity.

**Conclusion:**

The result indicated that *Silene macrosolen* has a significant antimalarial activity, justifying the traditional use of the plant material for treatment of malaria.

## 1. Introduction

Malaria is a fatal infectious disease that has a large burden of disease [1]. Malaria infects 219 million people globally with annual death of almost half a million [2, 3]. Sub-Saharan African countries including Ethiopia are disproportionately affected by malaria morbidity and mortality, with the highest burden on children under five and pregnant women [3, 4].

The rapidly evolving antimalarial drug resistance also poses a significant challenge in reducing the burden of malaria. Resistant parasites have been reported against all the available antimalarial medications [5, 6]. This indicates malaria may cause unprecedented morbidity and mortality in the future, justifying the need for new antimalarial drug development [5, 7].

Plants have been an important source of drugs for many centuries [8]. According to the WHO report, about 80% of Asians and Africans still rely on herbal medicine to meet their primary health-care needs [9, 10]. Most of the currently available antimalarial drugs also have a plant origin [8, 11]. Therefore, plants are considered as a robust source for the event of future effective antimalarial agents. Ethiopia is rich in biodiversity, and the traditional medicinal plant has played a significant role in malaria treatment [1, 12, 13].

The genus *Silene* is small shrubs containing about 700 species that belong to the Caryophyllaceae family. It has several reported pharmacological activities including antimicrobial, antitumor, phagocytic, antiviral, antifungal, and antioxidant activities [14].

*Silene macrosolen*, known by its vernacular name *saerosaero* (Tigrigna) and *wogert* (Amharic), is an herbal plant belonging to the Genus *Silene* that grows in different areas of Ethiopia [15]. Traditionally, it is used for treatment of malaria in Ethiopia [15, 16]. *S. macrosolen* is also used as a mosquito repellent and for treatment of an evil eye [17], hepatitis [18], and hemorrhoids [15]. However, pharmacological activity against malaria is not scientifically supported. Hence, the aim of this study was to evaluate *in vivo* antimalarial activity of the crude extract and solvent fractions of *S. macrosolen* using 4-day suppressive, prophylactic, and curative tests in *Plasmodium-berghei-*infected mice.

## 2. Materials and Methods

### 2.1. Chemicals, Reagents, and Drugs

Absolute methanol (Loba Chemic Pvt. Ltd., India), chloroform (Nice Chemicals Pvt. Ltd., India), ethyl acetate (Research-Lab Fine Chem Industries, India), Tween 80 (Loba Chemic Pvt. Ltd., India), microscopic oil immersion (Laboratory reagents, Ethiopia), Geimsa stain (Addis Ababa, Ethiopia), trisodium citrate (Loba Chemic Pvt. Ltd., India), and chloroquine phosphate (APF, Ethiopia) were used in this study, and the chemicals and reagents were laboratory or analytical grade.

### 2.2. Plant Material Collection and Authentication

Fresh roots of *S. macrosolen* were collected from Kilte Awulaelo District, Tigray, Ethiopia, located 825 km north of Addis Ababa. The collected plant material was identified by a botanist (name), and the specimen was deposited at the Biology Department, Gondar University, with voucher number 001GHA/2019.

### 2.3. Extraction and Fractionations of Plant Material

The collected root of the plant was cleaned with tap water and air dried under shade at room temperature before it was powdered using an electric mill. Extraction of the powdered root was carried by the maceration technique. Specifically, 500 grams of the powdered root was mixed with 2500 ml of 80% methanol that corresponded to one ratio five of between the weight of the powder and solvent proportion. The maceration continued for three days with intermediate shaking. The macerate was filtered using muslin cloth and Whatman filter paper (No. 1) consecutively, while the marc was remacerated twice with 80% methanol using the same procedure [19]. The combined filtrate was dried in an oven at around 40^0^C.

For fractionization, the dried crude extract (160 mg) was dissolved in distilled water and partitioned three times with an equal volume of chloroform. The chloroform layer was collected in a beaker and dried in the oven at around 40°C after combining together, while the aqueous layer was further shaken using equal volume of ethyl acetate three times following the same procedure as fractionation with chloroform. Finally, the aqueous fraction was taken and dried in an oven at around 40°C. The crude extract and the fractions were stored in the refrigerator throughout the experiment.

### 2.4. Experimental Animals and Parasite

Healthy Swiss albino mice of both sexes aged 6 to 8 weeks obtained from the Department of Pharmacology and Toxicology, School of Pharmacy, Mekelle University, were used to test the antimalarial activity of both the crude extract and solvent fractions. The mice were acclimatized for seven days prior to the experiments and housed in standard plastic cages with exposure to 12 h light/dark cycle and room temperature. The mice had free access to standard pellet and water *ad libitum* throughout the experiment. The mice were handled according to the internationally accepted laboratory animal use and care guideline [20]. The experimental animal studies were approved by the Institutional Review Committee of the College of Health Sciences, Mekelle University (ERC/1544/2018), before the actual commencement of the study.

Chloroquine-sensitive *P. berghei ANKA* strain obtained from Ethiopian Public Health Institute (EPHI), Addis Ababa University, was used to infect the mice. The parasites were maintained by passage of blood from infected to normal mice on a weekly basis.

### 2.5. Acute Oral Toxicity Test

An acute oral toxicity test was conducted in accordance to the Organization for Economic Co-operation and Development (OECD) 425 guidelines [21]. Accordingly, healthy and nonpregnant 5 female mice were used, and the first mouse was given a limited dose of 2000 mg/kg orally with an oral gavage after it was restricted from food access for 4 hours. The mouse was observed for 24 hours for any signs of toxicity and mortality. Afterward, the remaining 4 mice were treated with the same dose and observed strictly for the first one hour and intermittently for the subsequent three hours for any signs of toxicity like diarrhea, loss of appetite, hair erection, lacrimation, convulsions, salivation, lethargy, and paralysis. Follow-up was continued once daily for 14 days.

### 2.6. Grouping and Dosing

In each of the test models (4-day suppressive test, prophylactic test, and curative test), thirty mice were divided randomly into five groups, consisting of six mice.

The groups were categorized as groups I (treated with vehicle) and II (treated with chloroquine phosphate). The third, fourth, and fifth groups (group III–V) were treated with three different oral doses of the crude extract (Table 1) or solvent fractions.

### 2.7. Inoculation of Parasite

Mice with a parasitemia level of approximately 30% were used as a donor of chloroquine-sensitive strain of *P. berghei* ANKA throughout the experiment [22]. The donor mice were sacrificed by decapitation, and blood was taken by severing the jugular vein [23]. Blood was collected from all the donor mice, and it was diluted with 0.9% normal saline to obtain uniform 5 × 10^7^*P. berghei-*infected red blood cells (IRBC) in 1 ml of blood [24]. In each of the antimalarial model used, each mouse was inoculated with 0.2 ml of aliquot that contains 1 × 10^7^*P. berghei-*infected erythrocyte intraperitoneally [25].

### 2.8. *In Vivo* Antimalarial Activity Screening

#### 2.8.1. Four-day Suppressive Test

The 4-day suppressive test was used to measure the schizonticidal activity of the crude extract and fractions against *P. berghei*-infected mice [26]. Accordingly, thirty mice were inoculated with *P. berghei* on day 0 and randomly divided into five groups. The mice received different doses of the crude extract or solvent according to the grouping and dosing protocol described in Table 1. Treatment was continued for 4 days from day 0 to day 3, and on the 5^th^ day, a thin blood film was prepared from each mouse by taking blood through tail snip to determine the parasitemia level and percentage of inhibition. Rectal temperature, body weight, and packed cell volume (PCV) were recorded at day 0 (before parasite inoculation) and day 4.

#### 2.8.2. Prophylactic Activity (Peter's Repository Test)

Evaluation of the prophylactic potential of the crude extract was carried out according to the method described by Peters in 1975 [26]. Accordingly, 30 mice were randomly distributed into 5 groups **(**Table 1). The mice were treated daily for four consecutive days. On the 5^th^ day, the mice in all groups were infected with inoculums of 1 × 10^7^*P. berghei*-infected erythrocyte. On the 8^th^ day, blood smears were prepared from each mouse and the parasite level was determined. Body weight, temperature, and PCV were recorded at day 5 before infection and at the end of treatment.

#### 2.8.3. Test on Established Malaria Infection (Rane's Test)

The curative potential of the crude extract was also evaluated using the method described by Ryley and Peters [27]. In this experiment, 30 mice were inoculated with an inoculum of 1 × 10^7^*P. berghei-*infected erythrocyte intraperitoneally. Seventy-two hours after infection, the mice were randomly divided into 5 groups (Table 1) and treated daily for 4 days. A Giemsa-stained thin blood film was prepared from the tail of each mouse at the 4^th^ day (before the first dose) and on the 8^th^ day (24 hours after the last dose) to determine the parasitemia level. The body weight, packed cellular volume (PCV), and temperature of the mice were also recorded on the 4^th^ (before the first dose) and 7^th^ days (24 hours after the last dose).

### 2.9. Mean Survival Time

Mortality of each mouse in three of the models was monitored every day for a month, and the number of days from inoculation of the parasite up to death was recorded to evaluate the effect of the crude extracts and solvent fractions on survival time. The Mean Survival Time (MST) for each mouse was calculated using the following formula [8].

(1) $$\begin{array}{c} \text{MST}=\frac{\text{Sum of the survival time of mice in a groupdays}}{\text{Total number of mice in that group}}. \end{array}$$

### 2.10. Parasitemia Level

In all models, thin smears of blood film were made on coded microscope slides by taking blood from the tail of each mouse. The smears were fixed using absolute methanol and stained with 10% of Giemsa stain for 15 minutes [24]. The slides were then washed using tap water and dried at room temperature. The numbers of parasitized red blood cells were examined under a light microscope using an oil immersion objective of ×100 magnification. After microscopic evaluation of the parasitized red blood cells, the percentages of parasitemia and parasitemia suppression were calculated [8].

(2) $$\begin{array}{c} \%\text{ Parasitemia}=\frac{\text{Number of parasitized red bloodcells}}{\text{Total number of red bloodcells}}\times 100\%,\\ \%\text{ Suppression}=\left[\frac{A-B}{A}\right]\times 100, \end{array}$$

where *A* is the mean percentage of the parasitemia level of the mice in negative controls while *B* is the mean parasitemia level of mice in the treatment groups.

### 2.11. Packed Cell Volume

Blood samples were collected from the tail of each mouse using heparinized capillary tubes before and after treatment of the crude extract and fractions, and PCV was measured. The capillary tubes were filled with blood and sealed and centrifuged for 5 minutes at 10,000*g*. PCV was then calculated using a microhematocrit reader that takes the following formula into consideration [28]:

(3) $$\begin{array}{c} \text{PCV}=\frac{\text{Volume of erythrocytes in a given volume of blood}}{\text{Total blood volume}}\times 100. \end{array}$$

#### 2.11.1. Determination of Body Weight and Temperature Change

Mean percentage changes in body weight and rectal temperature before and after treatment were measured and recorded [12, 22].

### 2.12. Phytochemical Screening

The presence of different secondary metabolites such as flavonoids, alkaloids, terpenoids, tannins, saponins, and steroids in the crude extract and solvent fractions of *S. macrosolen* root was tested according to previously established protocols [29].

### 2.13. Data Management and Analysis

The data were entered into Statistical Package for Social Sciences (SPSS) version 23. The results were summarized as mean ± standard error of mean (SEM). One-way analysis of variance (ANOVA) followed by Tukey's post hoc test was used to compare differences in mean among the groups. A *P* value of less than 0.05 was considered statistically significant.

## 3. Results

### 3.1. Percentage Yield of the Plant Material

The percentage yield obtained from the *S. macrosolen* crude extract was 28% *w/w* with an actual yield of 420 mg. The highest percentage yield of the solvent fraction was obtained from the aqueous fraction (93.70%), while the lowest yield was from the chloroform fraction (1.25%) (Table 2).

### 3.2. Acute Toxicity Study

The acute toxicity study revealed the safety of the crude extract and solvent fractions at a dose of 2000 mg/kg. No gross behavioral and physical change and any sign of toxicity such as appetite loss, lacrimation, tremors, salivation, hair erection, and diarrhea were observed.

### 3.3. Antimalarial Studies

#### 3.3.1. Effect of the Crude Extract and Solvent Fractions of *S. macrosolen* on the 4-Day Suppressive Test

Mice treated with 200 mg/kg and 400 mg/kg doses of the crude extract showed a significant (*P* < 0.01 at 200 mg/kg and *P* < 0.001 at 400 mg/kg) level of parasite suppression compared to the negative control (Table 3). But none of the doses of the extract completely cleared the parasite. Mice treated with 400 mg/kg also exhibited a significant parasite suppression compared to the mice which received the lowest two doses (*P* < 0.01).

In the solvent fractions, all tested doses of the aqueous fraction (*P* < 0.05 at 100 and *P* < 0.01 at 200 and 400 mg/kg), 400 mg/kg dose of the chloroform fraction, and 200 and 400 mg/kg doses of the ethyl acetate fraction showed significant (*P* < 0.05) parasitemia suppression compared to the negative control. The highest percentage of chemosuppression of the solvent fractions was observed in the aqueous fraction (43.07%) followed by the ethyl acetate fraction (42.6%) and chloroform fraction (38.38%).

With regard to MST, the crude extract at 200 mg/kg (*P* < 0.05) and 400 mg/kg (*P* < 0.01) significantly increased the survival time of mice. In the solvent fractions, mice treated with 200 and 400 mg/kg doses (*P* < 0.01) of aqueous, chloroform, and ethyl acetate fractions and standard drug (*P* < 0.001) significantly increased mean survival time when compared to the negative control (Table 3).

The middle and highest doses of the crude extract resulted in lower body weight loss associated with infection compared to the negative control (*P* < 0.001). In the case of the fractions, 200 and 400 mg/kg (*P* < 0.05) doses of the aqueous fraction, 200 (*P* < 0.05) and 400 (*P* < 0.001) mg/kg doses of the chloroform fraction, and 200 (*p* < 0.01) and 400 (*P* < 0.05) mg/kg doses of the ethyl acetate fraction significantly prevented infection-induced body weight loss compared to the negative control. A significant weight increment was observed among chloroquine-treated groups (*P* < 0.001) compared to both the fraction- and vehicle-treated groups (Table 4).

As indicated in Table 4, analysis of percentage change in rectal temperature also revealed that mice treated with 200 (*P* < 0.05) and 400 (*P* < 0.001) mg/kg doses of the crude extract significantly prevented temperature reduction compared to the untreated group. In the solvent fractions, 200 (*P* < 0.05) mg/kg doses of the aqueous fraction as well as the intermediate and highest doses (*P* < 0.05) of the ethyl acetate fraction attenuated infection-induced reduction in rectal temperature compared to their respective negative control. The effects are comparable with the standard drug.

**Figure 1:**
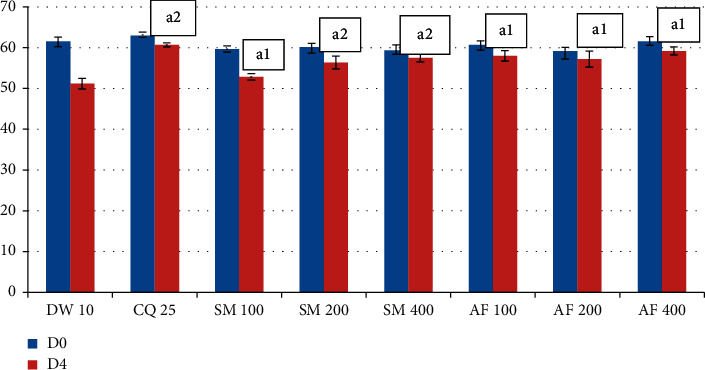
Effect of the crude extract and aqueous fraction of *S. macrosolen* on PCV of. *P. berghei*-infected mice in the 4-day suppressive test. Data are expressed as mean ± SEM (*n* = 6); CQ = chloroquine; DW = distilled water; AF = aqueous fraction; SM = *Silene macrosolen*; D0 = day 0; D4 = day; a = compared to the negative control; 1 = *P* < 0.05; 2 = *P* < 0.001.

**Figure 2:**
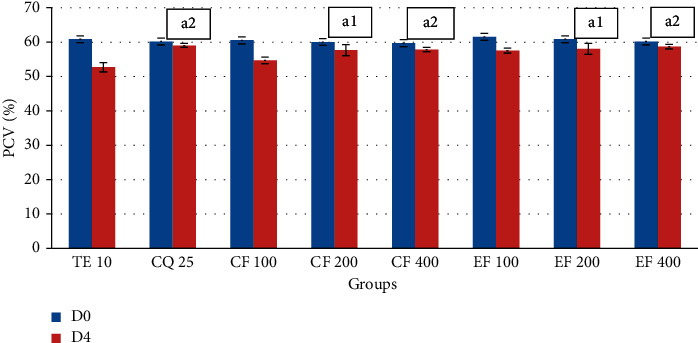
Effect of chloroform and ethyl acetate fractions of the *S. macrosolen* extract on PCV of *P. berghei*-infected mice in the 4-day suppressive test. Data were expressed as mean ± SEM (*n* = 6); CQ = chloroquine; TE = 2% Tween 80 (negative control for CF and EF fractions); CF = chloroform fraction; EF = ethyl acetate fraction; D0 = day 0; D4 = day 4; a = compared to the negative control; 1 = *P* < 0.05; 2 = *P* < 0.01.

In case of PCV, the 200 and 400 mg/kg doses of the crude extract showed comparable activity with the standard drug in preventing reduction in PCV (*P* < 0.001) associated with malarial infection (Figure 1). All groups treated with the solvent fractions have also progressively improved PCV. The highest prevention in PCV was observed in the highest dose of the ethyl acetate fraction (Figure 2).The effect produced by the fractions is comparable with the standard drug.

#### 3.3.2. Effect of the Crude Extract of *S. macrosolen* on the Prophylactic Test

The percentages of inhibitions of the crude extract were 12.48%, 21.23%, and 31.02% in mice treated with 100, 200, and 400 mg/kg doses, respectively, and only the 400 mg/kg dose showed a significant (*P* < 0.05) level of parasite suppression compared to the negative control. However, there was no significant difference in mean survival time among the groups (Table 5).

The mean percentage change in body weight and rectal temperature between D4 and D7 is summarized in Table 6. Only the highest dose (400 mg/kg) significantly prevented the loss of body weight compared to the negative control (*P* < 0.001). The groups that received the different doses of the crude extract and the standard drug had also significantly lower reductions in temperature compared to the negative control.

**Figure 3:**
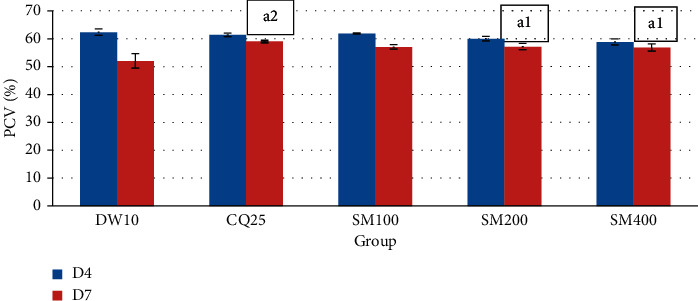
Effect of the crude extract of *S. macrosolen* on PCV of *P. berghei*-infected mice in the prophylactic test. Data are expressed as mean ± SEM (*n* = 6); CQ= chloroquine; DW = distilled water; SM = *Silene macrosolen*; D4 = day 4; D7 = day 7; a = compared to the negative control; 1 = *P* < 0.05; 2 = *P* < 0.01.

As indicated in Figure 3, all groups showed reduction in PCV. But, the middle and the highest doses of the crude extract (*P* < 0.05) and standard drug (*P* < 0.01) significantly prevented a reduction in PCV compared to the negative control.

#### 3.3.3. Effect of the Crude Extract of *S. macrosolen* on Rane's Test

In the curative test, the crude extract of *S. Macrosolen* had a considerable parasite suppressive activity against *P. berghei-*infected mice (Table 7). Although it is lower than the positive control (*P* < 0.001), all the evaluated dose levels showed a significant activity in parasite suppression compared to the negative control group. There was a significant difference in parasitemia suppression among 400 mg/kg- (*P* < 0.01) and 100 mg/kg-treated groups.

As indicated in Table 7, the two higher doses of the crude extract exhibited a significant increment in survival time as compared to the negative control (*P* < 0.01). The 200 and 400 mg/kg (*P* < 0.05) doses of the crude extract also significantly prevented body weight loss and temperature reduction as compared to the negative control (Table 8).

**Figure 4:**
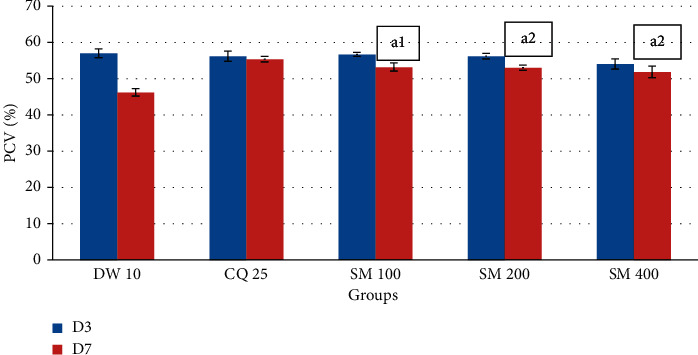
The effect of the crude extract of *S. macrosolen* on PCV of *P. berghei*-infected mice in Rane's test. Data are expressed as mean ± SEM (*n* = 6); CQ = chloroquine; DW = distilled water; SM = *Silene macrosolen*; D3 = day 3; D7 = day 7; a = compared to the negative control; 1 = *P* < 0.01; 2 = *P* < 0.001.

As indicated in Figure 4, PCV determination in the curative test revealed that 100 (*P* < 0.01), 200, and 400 mg/kg (*P* < 0.001) doses of the crude extract and chloroquine (*P* < 0.001) significantly prevented the reduction in PCV compared to the negative control.

### 3.4. Phytochemical Screening

Result of phytochemical screening revealed that the crude extract and solvent fractions of *S. macrosolen* contain secondary metabolites such as alkaloids, saponins, phenols, terpenoids, and flavonoids. However, tannins and anthraquinones were absent from the crude extract and solvent fractions of the plant.

## 4. Discussion

Developing new drugs is absolutely critical to fight against the challenge in malaria treatment. Herbal products have an undeniable situation in the development of antimalarial chemotherapeutic drugs. Along these lines, plants are considered as a solid hotspot for the advancement of future compelling antimalarial operators.

In the acute oral toxicity test in this study, any sign of toxicity and mortality was not observed. This indicates that the lethal dose (LD_50_) of the plant extract could be higher than 2000 mg/kg body weight as per the OECD guideline No. 425 [21].

Even though *P. berghei* is not exactly similar to that of the human *Plasmodium* parasites, it is the first step to screen *in vivo* antimalarial activities of test substances. Moreover, several conventional antimalarial drugs have been identified using this rodent malaria parasite. Chloroquine was used as a reference drug in the present study because the parasite used in this study is chloroquine sensitive [24, 28].

Models such as the 4-day suppressive test, prophylactic test, and Rane's test were used to investigate the antimalarial effect of *S. macrosolen*. In all these three models, the determination of percentage inhibition of the parasite is the most reliable parameter [13, 24].The efficacy of a compound is assessed by comparison of the parasitemia level and MST between treated and untreated mice [28]. PCV, body weight change, and change in body temperature are the other parameters used. An ideal antimalarial agent is expected to prevent the reduction in PCV, bodyweight loss, and reduction in rectal temperature in infected mice due to the rise in parasitemia [3].

As it can be seen from the results, the two higher doses of *S. macrosolen* in the crude extract significantly (*P* < 0.01 at 200 mg/kg and *P* < 0.001 at 400 mg/kg) suppressed the parasitemia level compared to the negative control in the 4-day suppressive test. The 39.23% parasite suppression exerted by 400 mg/kg in this test is comparable with another study [30] which has shown 40.73% parasitemia suppression. This shows the potential of the plant extract to prevent the increment in the parasite load in the blood. The antiplasmodial activity seen might be also due to the immunomodulatory effect of the plant extract [28, 31].

In the prophylactic test, only the 400 mg/kg dose showed a significant parasite suppressive activity (*P* < 0.05) against *P. berghei* infection. This might be due to the accumulation of the active constituent in the highest dose. Similarly, the crude extract also exerted a significant parasite suppression in Rane's test. Rane's test is a standard test commonly used for antimalarial screening. It relies on the ability of the standard inoculum of *P. berghei* to kill the recipient mouse thereafter.

In this model, the highest percentage of suppression was 35.82, at the 400 mg/kg (*P* < 0.001) dose level. This is comparable with another study [23] which has shown 35.79% parasite suppression. The parasitemia suppressive effect of the crude extract in all the three models increased with doses. This might be due to the higher concentration of the active secondary metabolites in higher doses [13].

As discussed above, the crude extract exhibited the highest chemosuppressive effect in the 4-day suppressive test with the lowest effect in the prophylactic test. The lower chemosuppressive effect in the prophylactic test might be due to the rapid clearance of the active component since it was given before infection at which the metabolic rate of the mice is active [32]. This finding is similar to other studies in which 4-day suppressive tests have a higher chemosuppressive effect than curative tests and prophylactic tests [22, 25].

The antimalarial effect of *S. macrosolen* seen may be due to the presence of alkaloids, saponins, phenols, terpenoids, and flavonoids. This is in agreement with other works in which the antiplasmodial activity of alkaloids [17], saponins [13], phenols [27], terpenoids [31], and flavonoids [33] has been reported. Phenolic compounds, flavonoids, alkaloids, and terpenoids have an antioxidant or free-radical scavenging effect, so these phytochemicals can prevent oxidative stress induced by the parasite [34]. Antioxidants can also inhibit heme polymerization, and unpolymerised heme is toxic for the parasite [35]. The proved immunomodulatory effect of terpenoids and flavonoids also might have an impact on the host-parasite interrelationship [34].

The three fractions of *S. macrosolen also* showed a parasite-suppressive effect with varying degree of suppression. The aqueous fraction **(**43.07%) and ethyl acetate fractions (42.61%) were found to possess higher blood schizontocidal activity than the chloroform fraction (38.38%). This could be attributed to the high concentration of phytochemicals in the aqueous fraction and the least number of phytochemicals in the chloroform fraction. The results are similar to former studies in which the aqueous fraction had higher activity than chloroform and ethyl acetate fractions [2, 31].

*In vivo* antiplasmodial activity can be classified as moderate, good, and very good if an extract displayed a percent parasite suppression equal to or greater than 50% at a dose of 400, 200, and 100 mg/kg, respectively [13, 34]. The determination of percentage inhibition of parasitemia is the most reliable parameter in antimalarial screening. Therefore, it is clear from the result (Table 3) that the *P. berghei*-infected mice treated with the crude extract of *S. macrosolen* reduced in percentage parasitemia compared to those of the untreated control animals.

Based on this classification, the crude extract and solvent fraction showed moderate antimalarial activity. Furthermore, a test substance is considered as active when it shows percentage suppression of parasitemia greater than 30% and prolongs survival time of infected mice [17, 22]. This result has supports of our study.

Regarding MST, the crude extract in the 4-day suppressive and curative tests increased survival time of mice significantly (*P* < 0.01) as compared to the negative control. On the other hand, the survival time did not increase significantly in the prophylactic test. The difference in survival time in the three models could be due to the differences in parasitemia inhibition [13, 36]. Similarly, the three fractions of *S. macrosolen* also increased the survival time of mice significantly compared to the negative control. Mice treated with the aqueous fraction lived a longer time followed by ethyl acetate and chloroform fractions. This might be due to the localization of active metabolites in the aqueous fraction [31]. Prolongation of the MST of mice indicates that the crude extract and solvent fractions suppressed *P. berghei* and reduced the pathologic effect of the parasite [37].

However, the crude extract in the three models and the solvent fractions in a 4-day suppressive test significantly attenuated body weight loss compared to their respective negative control. The improvement in body weight might be due to the parasite suppressive effect of the extract and solvent fractions. It might be also due to the effect of the crude extract and solvent fractions on the other aspects of malaria illness such as PCV and rectal temperature [23, 38].

Hypothermia is one of the manifestations of *P. berghei-*infected mice. The mechanism for fall in temperature may include the general debilitating effect of the infection, such as anemia, on heat production and/or heat conservation in small animals. Hypothermia in *Plasmodium berghei-*infected mice is also associated with increased turnover in brain serotonin, the neurotransmitter which reduces both food intake and body temperature in rodents [39].

Analysis of percentage change in rectal temperature in our study revealed that the mice treated with the crude extract and solvent fractions except in the prophylactic test and the chloroform fraction showed a significant prevention in the reduction of rectal temperature compared to their respective negative controls. The improvement in rectal temperature is probably due to a significant parasite-suppressive effect of an improvement in pathological conditions associated with parasite infection that causes a reduction in body temperature [13]. The observed antimalarial activity was dose dependent, but at a dose of 400 mg/kg body weight, a reduction in parasitemia was recorded with 35.82 percent chemosuppression on day 8 (Table 7) in Rane's test.

This may be due to the immunosuppressive activity of the extract. It had been reported that oral administration of phytochemicals such as saponin, tannins, and phenols possessed the ability to suppress cellular immunity [40].

Malaria-induced reduction in PCV is another manifestation of *P. berghei-*infected mice. It is caused by RBC destruction as a result of parasite multiplication, reduced erythropoiesis, and dyserythropoiesis [23]. PCV was measured to estimate the efficacy of the crude extract and solvent fractions in averting hemolysis due to rising parasitemia levels [34]. Analysis of the mean percentage change in PCV in this study revealed that the crude extract and solvent fractions significantly prevented a reduction in PCV as compared to their respective negative control. The relative reversal of the reduction in PCV might be due to the significant reduction in the parasite level of infected mice and/or prevention in hemolysis [13, 41]. In both the crude extract and solvent fractions, the highest improvement in PCV was observed in the higher doses. From the three models, the lowest reduction in PCV was seen in the 4-day suppressive test followed by curative test and prophylactic test. This effect in PCV agreed with the results of parasite suppression [12].

The potential of plant-derived natural products for antimalarial drug discovery has been examined in several review papers [42–44]. Previous studies have shown that plant-derived alkaloids have a great potential for antimalarial drug development [42–44]. Triterpenoid and steroid saponins have been found to be detrimental to several infectious protozoans such as *Plasmodium falciparum* [45].The antimalaria activity of *S*. *macrosolen* might be due to the presence of one of these constituents based on phytochemical analysis of the root of *S*. *macrosolen*. The results from our studies support the scientific credential to the folkloric use of the root extract of *S*. *macrosolen* for the treatment of malaria in Ethiopia.

## 5. Conclusions

The results of the present study confirmed that *S. macrosolen* is safe and possesses moderate antimalarial activity. The solvent fractions also showed an antimalarial activity with various degrees of chemosuppression with an aqueous fraction being relatively effective. Our results support the traditional use of plants in the treatment of malaria by traditional users in Ethiopia. In the future, subacute and chronic toxicity tests should be conducted to check its long-term safety profile. It is also important to identify and isolate active constituents that explain the observed antimalarial activity on the root of *S. macrosolen.*

## Figures and Tables

**Table 1:** Animal grouping and dosing.

Groups	Dose (mg/kg)	
	Crude extract	Fractions
I	10 ml/kg^*∗*^	10 ml/kg^*∗∗*^
II	25^*∗∗∗*^	25^*∗∗∗*^
II	100	100
IV	200	200
V	400	400

^*∗*^Distilled water. ^*∗∗*^3% tween 80 in the fractions. ^*∗∗∗*^Chloroquine phosphate.

**Table 2:** Percentage yield and physical properties of the crude extract and solvent fractions of *S. macrosolen*.

Extract/fractions	Nature of extract/fractions	Color of extract/fractions	Actual yield (g)	% yield (*w/w*)
Chloroform	Viscous	Brown	2	1.25
Ethyl acetate	Viscous	Brown	4	2.5
Aqueous	Solid powder	Whitish yellow	150	93.7
Crude extract	Solid powder	Whitish yellow	420	28

**Table 3:** Effect of the crude extract and solvent fractions of *S. macrosolen* on the parasite level, parasite suppression, and MST of *P. berghei*-infected mice in the 4-day suppressive test.

Group	Doses (mg/kg)	% parasitemia (*M* ± SEM)	% suppression	Mean survival time (days)
DW	10 ml/kg	54.82 ± 2.97	0	7.17 ± 0.31
CQ	25	0.00 ± 0.00^a3^	100^a3^	30.00 ± 0.00^a3^
SM	100	46.97 ± 0.97^b3e2^	14.33^b3e2^	7.67 ± 0.21
SM	200	42.78 ± 2.44^a2b3e1^	21.97^a2b3e1^	8.33 ± 0.33^a1^
SM	400	33.32 ± 2.08^a3b3c2d1^	39.23^a3b3c2d1^	8.67 ± 0.33^a2^
AF	100	38.48 ± 5.66^a1b3^	29.81^a1b3^	8.33 ± 0.33^b3^
AF	200	32.63 ± 3.23^a2b3^	40.48^a2b3^	9.00 ± 0.37^a2b3^
AF	400	31.21 ± 4.02^a2b3^	43.07^a2b3^	9.33 ± 0.42^a2b3^

TE	10 ml/kg	48.85 ± 7.34	0	6.67 ± 0.33
CQ	25	0.29 ± 0.12^a3^	99.41^a3^	30.00 ± 0.00^a3^
CF	100	41.79 ± 3.91^b3^	14.46^b3^	8.00 ± 0.52^b3^
CF	200	39.71 ± 4.10^b3^	18.72^b3^	8.17 ± 0.40^b3^
CF	400	30.10 ± 3.90^a1b2^	38.38^a1b2^	8.50 ± 0.34^a1b3^
EF	100	35.20 ± 3.19^b3^	27.95^b3^	8.50 ± 0.34^a1b3^
EF	200	29.46 ± 3.72^a1b2^	39.70^a1b2^	8.67 ± 0.42^a2b3^
EF	400	28.03 ± 4.61^a1b2^	42.61^a1b2^	9.00 ± 0.52^a2b3^

Data are expressed as mean ± SEM (*n* = 6); AF = aqueous fraction; CQ = chloroquine; DW = distilled water (negative control for the crude extract and AF fraction); TE = 2% Tween 80 (negative control for CF and EF fractions); CF = chloroform fraction; EF = ethyl acetate fraction; a = compared to the negative control; b = compared to the positive control; c = compared to 100 mg/kg crude extract; d = compared to 200 mg/kg crude extract; e = compared to 400 mg/kg crude extract; SM = *Silene macrosolen*; 1 = *P* < 0.05; 2 = *P* < 0.01; 3 = *P* < 0.001.

**Table 4:** Effect of the crude extract and solvent fractions of *S. macrosolen* on body weight and rectal temperature of *P. berghei*-infected mice in the 4-day suppressive test.

Groups	Weight (g)			Temperature		
	D0	D4	% change	D0	D4	% change
DW10 ml/kg	29.17 ± 0.60	24.73 ± 0.74	−15.2	36.35 ± 0.18	33.67 ± 0.37	−7.38
CQ 25	27.85 ± 0.85	28.25 ± 0.79	1.50^a3^	36.15 ± 0.08	36.07 ± 0.15	−0.23^a3^
SM100	28.67 ± 0.85	25.48 ± 0.72	−11.05^b3^	36.23 ± 0.22	33.73 ± 0.37	−6.90^b3^
SM200	29.45 ± 0.79	27.42 ± 0.68	−6.86^a3b3^	36.35 ± 0.09	34.87 ± 0.15	−4.07^a1b2^
SM400	29.33 ± 0.61	27.08 ± 0.72	−7.72^a3b3^	35.75 ± 0.31	34.88 ± 0.31	−2.42^a3^
AF100	28.13 ± 0.79	24.82 ± 0.72	−11.79^b2d1^	36.70 ± 0.35	34.98 ± 0.39	−4.67^b1^
AF200	27.00 ± 0.57	24.93 ± 0.65	−7.68^a2b2c1^	36.82 ± 0.46	35.68 ± 0.47	−3.08^a1^
AF400	27.68 ± 0.52	25.10 ± 0.54	−9.34^a2b2^	36.67 ± 0.20	35.12 ± 0.51	−4.21^b1^

TE10 ml/kg	27.60 ± 0.68	24.90 ± 0.68	−9.78	36.55 ± 0.13	34.45 ± 0.40	−5.75
CQ25	26.58 ± 0.45	28.33 ± 0.43	6.55^a3^	36.25 ± 0.21	36.40 ± 0.17	0.41^a3^
CF100	27.40 ± 0.77	25.27 ± 0.93	−7.79^b3d1^	36.47 ± 0.27	35.02 ± 0.15	−3.98^b1^
CF200	27.40 ± 0.77	26.20 ± 0.83	−4.38^a1b3^	36.63 ± 0.25	35.12 ± 0.29	−4.14^b2^
CF400	26.90 ± 0.55	26.18 ± 0.56	−2.65^a3b3c1^	36.83 ± 0.21	35.75 ± 0.40	−2.94
EF 100	27.12 ± 0.75	25.65 ± 0.94	−5.50^b3^	36.70 ± 0.31	35.15 ± 0.33	−4.22^b2^
EF200	27.20 ± 0.68	26.52 ± 0.94	−2.59^a2b3^	36.53 ± 0.33	35.97 ± 0.33	−1.55^a2^
EF400	27.73 ± 0.79	26.40 ± 0.73	−4.77^a1b3^	36.70 ± 0.19	36.17 ± 0.12	−1.45^a2^

Data are expressed as mean ± SEM (*n* = 6); CQ = chloroquine; DW = distilled water(negative control for the crude and AF fraction); SM= *Silene macrosolen*; D0 = day 0; D4 = day 4; a = compared to the negative control; b = compared to the positive control; c = compared to 100 mg/kg of CF; d = compared to 400 mg/kg of CF; AF = aqueous fraction; TE = 2% Tween 80 (negative control for CF and EF fractions); CF = chloroform fraction; EF = ethyl acetate fraction; 1 = *P* < 0.05; 2 = *P* < 0.01; 3 = *P* < 0.001.

**Table 5:** Effect of the crude extract of *S. macrosolen* on the parasite level, parasite suppression, and mean survival time of *P. berghei*-infected mice in the prophylactic test.

Group	Doses (mg/kg)	% parasitemia (*M* ± SEM)	% suppression	Mean survival time (days)
DW	10 ml/kg	55.71 ± 1.14	0	6.67 ± 0.33
CQ	25	0.38 ± 0.14^a2^	99.32^a2^	29.17 ± 0.83^a2^
SM	100	48.75 ± 4.89^b2^	12.48^b2^	7.33 ± 0.21^b2^
SM	200	43.88 ± 5.59^b2^	21.23^b2^	7.50 ± 0.22^b2^
SM	400	38.43 ± 3.93^a1b2^	31.02^a1b2^	8.33 ± 0.21^b2^

Data are expressed as mean ± SEM (*n* = 6); CQ = chloroquine; DW = distilled water; SM = S*ilene macrosolen*; a = compared to the negative control; b = compared to the positive control 1 = *P* < 0.05; 2 = *P* < 0.001.

**Table 6:** Effect of the crude extract of *S. macrosolen* on body weight and rectal temperature of *P. berghei*-infected mice in the prophylactic test.

Groups	Weight (g)			Temperature		
	D4	D7	% changes	D4	D7	% changes
DW10 ml/kg	28.27 ± 0.49	24.33 ± 0.30	−13.89	35.97 ± 0.16	33.07 ± 0.23	−8.06
CQ25	29.28 ± 0.72	28.52 ± 0.72	−2.52^a2^	36.23 ± 0.08	35.70 ± 0.18	−1.47^a2^
SM100	27.63 ± 0.80	24.80 ± 0.67	−10.22^b2^	36.15 ± 0.19	33.58 ± 0.15	−7.10^b2^
SM200	29.27 ± 1.06	26.23 ± 0.78	−10.24^b2^	36.37 ± 013	34.13 ± 0.33	−6.14^b2^
SM400	27.85 ± 0.71	25.78 ± 0.57	−7.35^a2b1^	36.48 ± 0.14	34.37 ± 0.28	−5.80^b2^

Data are expressed as mean ± SEM (*n* = 6); CQ = chloroquine; DW = distilled water; SM = *Silene macrosolen*; D4 = day 4; D7 = day7; a = compared to the negative control; b = compared to the positive control; 1 = *P* < 0.05; 2 = *P* < 0.001.

**Table 7:** Effect of the crude extract of *S. macrosolen* on the parasite level, parasite suppression, and mean survival time of *P. berghei*-infected mice in Rane's test.

Group	Dose (mg/kg)	% parasitemia (mean ± SEM)		% suppression	Mean survival time (days)
		D3	D7		
DW	10 ml/kg	49.89 ± 1.84	56.65 ± 3.19	0	6.17 ± 0.31
CQ	25	48.59 ± 2.25	0.03 ± 0.01^a3^	99.95^a3^	30.00 ± 0.00^a3^
SM	100	50.43 ± 3.29	45.20 ± 2.75^a2b3d1^	20.23^a2b3^	6.83 ± 0.31^b3^
SM	200	47.07 ± 1.49	43.41 ± 1.17^a2b3^	23.38^a2b3^	7.50 ± 0.22^a2b3^
SM	400	47.83 ± 0.99	36.36 ± 1.7^a3b3c1^	35.82^a3b3c1^	7.67 ± 0.21^a2b3^

Data are expressed as mean ± SEM (*n* = 6); CQ = chloroquine; DW = distilled water; D3 = day 3; D7 = day7; SM = *Silene macrosolen*; a = compared to the negative control; b = compared to the positive control; c = compared to 100 mg/kg crude extract; d = compared to 400 mg/kg crude extract; 1 = *P* < 0.05; 2 = *P* < 0.01; 3 = *P* < 0.001.

**Table 8:** Effect of the crude extract of *S. macrosolen* on body weight and rectal temperature of *P. berghei-*infected mice in Rane's test.

Groups	Weight (g)			Temperature		
	D4	D7	% changes	D4	D7	% changes
DW10 ml/kg	27.75 ± 0.31	24.15 ± 0.35	−12.98	35.48 ± 0.26	32.93 ± 0.21	−7.15
CQ25	27.17 ± 0.54	26.40 ± 0.60	−2.77^a3^	35.40 ± 0.19	34.75 ± 0.21	−1.84^a2^
SM100	27.83 ± 0.54	25.28 ± 0.48	−9.15^b1^	35.02 ± 0.26	33.52 ± 0.33	−4.26
SM200	27.12 ± 0.59	25.15 ± 0.71	−7.27^a1^	35.25 ± 0.18	34.10 ± 0.16	−3.26^a1^
SM400	26.77 ± 0.82	24.73 ± 0.91	−7.66^a1^	35.25 ± 0.19	34.28 ± 0.29	−2.73^a1^

Data are expressed as mean ± SEM (*n* = 6); CQ = chloroquine; DW = distilled water; SM = *Silene macrosolen*; D3 = day 3; D7 = day 7; a = compared to the negative control; b = compared to the positive control; 1 = *P* < 0.05; 2 = *P* < 0.01; 3 = *P* < 0.001.

## Data Availability

All the data used to support findings of this study are available with the corresponding author when requests come from concerned bodies.
